# A Single-Arm, Open-Label, Pilot, and Feasibility Study of a High Nicotine Strength E-Cigarette Intervention for Smoking Cessation or Reduction for People With Schizophrenia Spectrum Disorders Who Smoke Cigarettes

**DOI:** 10.1093/ntr/ntab005

**Published:** 2021-03-16

**Authors:** Pasquale Caponnetto, Jennifer DiPiazza, Jason Kim, Marilena Maglia, Riccardo Polosa

**Affiliations:** 1 Department of Educational Science, University of Catania, Catania, Italy; 2 Faculty of Health Science and Sport, University of Stirling, Stirling, UK; 3 Hunter Bellevue School of Nursing, Hunter College-City University of New York, New York, NY, USA; 4 Clinical and Translational Science Center, Weill Cornell Medicine, New York, NY, USA; 5 Centro per la Prevenzione e Cura del Tabagismo (CPCT), Azienda Ospedaliero-Universitaria “G.Rodolico-S. Marco”, Università di Catania, Catania, Italy; 6 Center of Excellence for the Acceleration of Harm Reduction (CoEHAR), University of Catania, Catania, Italy; 7 Department of Clinical and Experimental Medicine, University of Catania, Catania, Italy

## Abstract

**Introduction:**

An estimated 60%–90% of people with schizophrenia smoke, compared with 15%–24% of the general population, exacerbating the already high morbidity and mortality rates observed in this population.

**Aims and Methods:**

This study aimed to assess the feasibility of using a new-generation high strength nicotine e-cigarette to modify smoking behavior in individuals with schizophrenia spectrum disorders who smoke cigarettes. A single-arm pilot study was conducted with 40 adults with schizophrenia spectrum disorders who smoked and did not intend to reduce or quit smoking. Participants were given a 12-week supply of a JUUL e-cigarette loaded with a 5% nicotine pod. The primary outcome was smoking cessation at week 12. Additional outcomes included: smoking reduction, continuous abstinence at week 24, adoption rate, adherence to the e-cigarette, feasibility, acceptability, and subjective effects.

**Results:**

Sixteen (40%) participants quit by the end of 12 weeks. For the whole sample, we observed an overall, sustained 50% reduction in smoking or smoking abstinence in 37/40 (92.5%) of participants and an overall 75% reduction in median cigarettes per day from 25 to six was observed by the end of the 12 weeks (*p* < .001).

**Conclusions:**

A high strength nicotine e-cigarette has the potential to help people with schizophrenia spectrum disorders to quit or reduce smoking. Further research with a larger sample and a comparator group is needed. The results provide useful information and direction to augment the existing body of knowledge on smoking cessation for people with schizophrenia spectrum disorders.

**Implications:**

Considering that most people with schizophrenia spectrum disorders continue smoking, alternative and efficient interventions to reduce or prevent morbidity and mortality are urgently needed. This study showed that adults who smoke and were not motivated to quit, when provided a new-generation e-cigarette with high nicotine content, demonstrated substantially decreased cigarette consumption without causing significant side effects. Although not specifically measured in this study, nicotine absorption in new-generation devices has been shown to be consistently superior compared with the first generation of e-cigarette devices, and this may help explain the lower quit rates in studies using earlier generation devices.

## Introduction

An estimated 60%–90% of people with schizophrenia smoke, compared with 15%–24% of the adult general population^1–7^; this group smokes more heavily and is more dependent on tobacco than those without mental illness.^8^ Individuals with schizophrenia extract more nicotine from cigarettes compared with those without mental illness and have higher blood levels of nicotine after smoking.^9^ Nicotine’s addiction liability is confirmed by the high smoking rates and low quit success rates observed despite its well-known adverse impact on health.^10^

Williams et al.^11^ measured serum nicotine levels and ad libitum smoking for 24 + 2 hours using a smoking topography device in 75 people with schizophrenia who smoked cigarettes compared with 86 people without mental illness who smoked. People with schizophrenia smoked more cigarettes, took more frequent puffs, took less time to smoke a single cigarette, and had a higher nicotine intake than those without mental illness who smoked.

As a result of high smoking rates, people with a mental health condition also have high rates of morbidity and mortality compared with the general population.^12,13^ Therefore, quitting smoking is particularly important for this group.

In a meta-analysis of two randomized controlled trials (RCTs) that examined the effect of e-cigarettes for smoking cessation in general population samples,^14,15^ participants using an e-cigarette were more likely to have abstained from smoking for at least 6 months compared with those using a placebo e-cigarette.^16^ Other systematic reviews and meta analyses of the effect of e-cigarette on smoking cessation have reported that e-cigarettes have a negative effect on cessation^17^ or were inconclusive.^18,19^ The discrepancies in findings across several systematic reviews may relate to variability in types of studies, participants, outcomes, and length of follow-up included in each meta-analysis.^20^ A more recent RCT found that e-cigarettes were almost twice as effective for smoking cessation than nicotine-replacement therapy.^21^

The number of studies examining e-cigarettes for smoking cessation in the general population is now fairly substantial but yields mixed conclusions. While far fewer studies have been conducted with people with schizophrenia, emerging research suggests that e-cigarettes may be useful for smoking reduction in people with schizophrenia spectrum disorders. In the first published study of e-cigarettes as a potential cessation or reduction aid for adults with schizophrenia who smoked cigarettes, 14 patients not motivated to stop smoking were provided with a rechargeable e-cigarette kit and a 12-week supply of nicotine cartridges and advised to use the product ad libitum.^20^ The e-cigarette was a first-generation “Categoria” e-cigarette with replaceable 7.4 mg/mL nicotine cartridges. At 12-month follow-up, 50% of people with schizophrenia who smoked cigarettes had reduced their cigarettes per day (CPD) by 50%, and a further 14% had quit smoking completely with no increase in psychiatric symptoms.^22^

Subsequently, Pratt et al.^23^ enrolled 21 outpatients with severe mental illness (schizophrenia, schizoaffective, or bipolar disorder) not motivated to quit who smoked at least 10 CPD. Participants were given a 4-week supply of a second-generation e-cigarette based on each participant’s level of use of cigarettes, and they were evaluated weekly for 1 month. Nineteen participants completed the study. The study found a significant reduction in smoking with a mean self-reported decline in use of cigarettes from 192 to 67 cigarettes per week confirmed by carbon monoxide expired air (CO) reduction from 27 to 15 ppm.

In a more recent study, Hickling et al.^24^ conducted a 24-week pilot study to investigate the efficacy and acceptability of a 6-week supply of a first-generation e-cigarette to reduce smoking in 50 people with severe mental illness who smoked cigarettes and were not motivated to quit, including 42 (84%) participants with schizophrenia spectrum disorders. These participants were provided with NJOY disposable e-cigarettes with 45 mg/mL nicotine and were encouraged to replace cigarettes with e-cigarettes as much as possible. At the end of the 6-week supply phase, 37% of participants had reduced their tobacco consumption and 7% had stopped smoking. Four weeks after the end of the 6-week supply, 26% of participants had reduced their tobacco consumption and 5% had quit combustible cigarettes. At final follow-up (24 weeks), 25% of participants had reduced their tobacco consumption and 2% had quit smoking cigarettes.

The efficiency of nicotine delivery among e-cigarettes has improved substantially since first marketed more than 10 years ago and there is a growing consensus that these products are significantly less harmful than combustible cigarettes.^20,25–27^ Recent research indicates that nicotine pharmacokinetics of the JUUL e-cigarette with 5% nicotine strength (a device that utilizes a nicotine salt formulation) approximates the nicotine delivery of combustible cigarettes.^28–30^ The 5% nicotine strength product is far more efficient in delivering nicotine compared with the pod with 1.5% nicotine strength.^31^

The aim of this study was to evaluate the feasibility of modifying smoking behavior in people with schizophrenia spectrum disorders who were not interested in giving up smoking, by providing them with a JUUL e-cigarette starter kit and replaceable pods with 5% nicotine strength for 12 weeks. We also assessed changes in mental health symptoms, body weight, subjective effects of the use of the e-cigarettes, and recruitment and retention rates. We considered using other e-cigarette devices and nicotine strengths but chose the JUUL for its specific characteristics to efficiently deliver high strength nicotine. Given the very limited published literature on the use of e-cigarettes for smoking reduction or cessation in people with schizophrenia, we conducted a single-arm pilot study to inform the parameters for a future RCT.

## Methods

### Design

This was a single-arm, open-label pilot study to observe combustible cigarette use behavior in adults with a schizophrenia spectrum disorder diagnosis who smoked, who did not intend quit smoking, and who were invited to use an e-cigarette. The e-cigarettes were offered to participants as an alternative to cigarettes. The design was informed by a conceptual framework for defining feasibility and pilot studies developed by Eldridge et al. They argue that pilot studies are a subset of feasibility, rather than the two being mutually exclusive,^32^ and that single-arm pilot studies (as in the current research) can still answer feasibility questions. Therefore, this single-arm pilot study aimed to determine if and how the proposed intervention could be delivered in practice and how to proceed with testing the intervention in a future, larger study.

### Participants

Forty participants were recruited between September and October 2017. We wrote to physicians, psychiatrists, and other health care providers to inform them about the study. Flyers were posted within and outside of the Smoking Cessation Center of Catania University (Centro per la Prevenzione e Cura del Tabagismo—CPCT), at the Policlinico Vittorio Emanuele. Participants were recruited from Catania outpatient psychiatric clinics by researchers of CPCT. Clinicians from outpatient psychiatric clinics identified suitable participants and drew their attention to the study flyers.

As this was a single-arm pilot study, a formal sample size calculation was not required. However, with approximately 40 participants in the study, a 95% confidence interval for the proportion of study participants with self-reported complete cessation from combustible tobacco cigarettes at each follow-up visit could be constructed to be within ±17.6% of the true proportion of people who successfully quit. This calculation, performed during the protocol study design, was based on a previous study about the impact of e-cigarettes on smoking reduction and cessation in adults with schizophrenia who smoked,^22^ which assumed that approximately 20% of participants would have achieved complete cessation from tobacco cigarette smoking during the entire study period. Up to 40 participants were recruited at baseline (BL), as this study assumed a conservative attrition rate of 50%. Primary and secondary outcome measures were analyzed by including all enrolled participants assuming, on the basis of the intention-to-treat principle, that all individuals lost to follow-up would be classified as continuing to smoke.^33^

Inclusion criteria were:

1. Adults attending psychiatric outpatient clinics in Catania who smoked 20 or more cigarettes daily were included. The rationales for these criteria were the opportunities, to study a potentially effective harm reduction alternative for heavy smokers who were cigarette dependent and who had difficulties to quit and to compare the results to those of a previous study that enrolled heavy smokers with schizophrenia spectrum disorders who smoked 20 or more combustible cigarettes.^22^
2. Aged 21–65 years.
3. Not intending to reduce or quit smoking.
4. Able to meet the criteria for a schizophrenia spectrum disorder diagnosis without evidence of current exacerbation of illness, defined as “no relapse to hospitalization within the last three months and no change in antipsychotic medication within the last month.”  ^34^ In terms of this last inclusion criterion, a clinical psychologist or a psychiatrist not involved in the study made the diagnosis based on criteria from the DSM-V.^35^

Exclusion criteria were: (1) pregnancy, (2) breastfeeding, (3) myocardial infarction or angina pectoris within the past 3 months, (4) current poorly controlled asthma or chronic obstructive pulmonary disease, (5) use of smokeless tobacco or any other tobacco products, and (6) use of nicotine-replacement therapy or other smoking cessation therapies within the last 3 months.

### Intervention

At the BL visit, participants were given a free e-cigarette starter kit containing one JUUL device with a charger and 5% nicotine pods, Virginia tobacco flavor with instructions on how to charge, activate, and use the e-cigarette. A 4-week supply of pods equivalent to their current cigarette smoking behavior, according to the manufacturer’s guidelines, was supplied to each participant (one pod for every packet of 20 cigarettes; mean 128, minimum 80, and maximum 200). Eligible participants were invited to use a JUUL e-cigarette for at least 12 weeks and were followed up prospectively for 24 weeks. Participants received a 4-week supply of pods on three occasions, BL, week 4 (study visit 2), and week 8 (study visit 3). Participants were informed verbally and in writing through the patient information sheet and consent form, that the product was potentially less harmful than combustible cigarettes and could be used as a cigarette substitute as much as they liked. Researchers could be contacted by phone if participants needed technical and medical assistance. Limited behavioral support was provided as part of the intervention and included behavior substitution of combustible cigarettes with e-cigarettes and self-monitoring of combustible cigarette consumption through the use of study diaries.^36^

**Figure 1.**
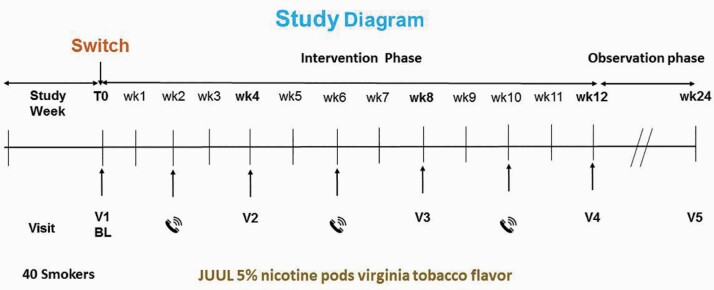
Study diagram. BL = baseline.

Participants attended a total of five study visits at the smoking cessation clinic, University of Catania (Figure 1).

The device used in the study—JUUL is an e-cigarette that was created by PAX Labs and is a closed pod e-cigarette product. The pod contains 0.7 mL of e-liquid and up to 5% nicotine by weight. The e-liquid composition includes propylene glycol, glycerol, nicotine, benzoic acid, and flavoring. JUUL is breath actuated, has no user modifiable settings, and is a temperature-regulated e-cigarette resembling a USB flash drive. The battery (200 mAh) is not removable and incorporates battery regulation features. The pod contains nicotine salts that are absorbed more readily into the bloodstream, are tolerated by the airway epithelium, and are able to deliver high concentrations of nicotine at a rate similar to cigarettes.^28,29,37^

The e-cigarettes used in the study were donated by the manufacturer, PAX Labs (on June 13, 2017 the company became known as JUUL Labs). At the time the research was conducted JUUL Labs were not part owned by Altria, a tobacco company. Due to the lack of availability at that time in Italy, PAX Labs (at that point renamed JUUL Labs) also agreed to supply cartridges/pods for a further 3 months after the end of the pilot to participants who expressed a wish to continue using them. No separate funding was secured for the study. Altria Group (formerly Philip Morris Companies) acquired a 35% stake in JUUL Labs on December 20, 2018 but the study was completed before Altria invested in JUUL.

Ethical approval was obtained for both the Italian University of Catania and the University of Stirling ethics committee.

### Measures

The following information was recorded at BL: demographics, smoking history and pack-years, CO, vital signs and body weight, and mental health symptoms. At study visits 2 (week 4), 3 (week 8), participants were invited back for further assessments CO, vital signs and body weight, mental health symptoms, and subjective effects and to return unused study products, check their study diary, and receive another 4-week supply of pods. At study visit 4 (week 12), participants returned unused study products and their study diary was checked. At this visit the following data were recorded: number of cigarettes smoked, pods used daily since the last visit, CO, vital signs and body weight, mental health symptoms, and subjective effects. At study visit 5 (week 24), participants’ study diaries were checked. At this visit the following data were recorded: number of cigarettes smoked daily since the last visit, CO, vital signs, body weight, and mental health symptoms.

Cigarette dependence was measured by the Fagerstrom test for cigarette dependence.^38^ The self-report questionnaire consists of six questions. The scores obtained on the test permit the classification of cigarette dependence into three levels: mild (0–3 points), moderate (4–6 points), and severe (7–10 points). CO was measured by a portable device (Micro CO, Micro Medical Ltd, Kent, United Kingdom).

Smoking abstinence was measured by continuous abstinence rates (CARs), defined as the percentage of participants remaining continuously abstinent from week 9 to each in-clinic visit through week 24. CARs during weeks 9–12 were the primary endpoint and CARs during weeks 9–24 were one of the secondary endpoints. CARs were verified by self-report and study diaries and exhaled carbon monoxide (≤10 ppm) measurements.

In between study visits participants were asked to maintain a daily study diary recording product use, number of tobacco cigarettes smoked, and adverse events (AEs). Participants reported AEs from a defined list of side effects and were also asked to keep track of any AEs they felt may be associated with e-cigarette using the study diary. The diary was reviewed with the participant by the researcher at each study visit. Participants who did not complete their diary between study visits completed it during the study visit.

Mental health symptoms were measured with the Scale for the Assessment of Positive Symptoms (SAPS)^39^ and the Scale for the Assessment of Negative Symptoms (SANS),^40^ two of the most widely used scales to measure symptoms of schizophrenia.^41^ The SANS and SAPS are both used frequently in clinical and research settings and reliability and validity have been shown to be consistent in multiple cross-cultural settings.^42^ SAPS measures positive symptoms on a 34 item, 6-point scale; four domains (hallucinations, delusions, bizarre behavior, and positive formal thought disorder) are rated from 0 (absent) to 5 (severe). SANS measures negative symptoms on a 25 item, 6-point scale. Items are listed under the five domains of affective blunting, alogia, avolition/apathy, anhedonia/asociality, and attention and rated from 0 (absent) to 5 (severe).

Subjective effects were assessed by Modified Cigarette Evaluation Questionnaire (mCEQ) scoring that reported frequency and percent of participants reporting (1) enjoyment of respiratory tract sensations, (2) psychological reward, and (3) satisfaction, among users of e-cigarettes. The mCEQ has good reliability and validity^43^ and was used to examine subjective effects of e-cigarette use on respiratory tract sensations, acceptability of e-cigarettes as substitutes for combustible tobacco cigarettes, psychological reward, satisfaction, craving reduction, aversion, and enjoyment.

Previous studies have used this questionnaire for e-cigarettes.^44–46^ It is a self-administered questionnaire that contains 12 items covering both the reinforcing and the aversive effects of smoking. Participants were asked to rate the extent to which the e-cigarette they used was satisfying, tasted good, made them dizzy, calmed them down, helped them concentrate, made them feel more awake, reduced appetite, made them nauseated, decreased irritability, produced enjoyable sensations in the throat and chest, immediately reduced craving for cigarettes and was enjoyable to smoke. The items were rated on a 7-point scale of 1 (not at all), 2 (very little), 3 (a little), 4 (moderately), 5 (a lot), 6 (quite a lot), and 7 (extremely). The mCEQ uses three multi-item domains (subscales) and two single items: “Smoking Satisfaction” (items 1, 2, plus item 12, the range of scores is minimum 3, maximum 21); “Psychological Reward” (items 4–8, the range of scores is minimum 5, maximum 35); “Aversion” (items 9 and 10, the range of scores is minimum 2, maximum 14); “Enjoyment of Respiratory Tract Sensations” (item 3, the range of scores is minimum 1, maximum 7); and “Craving Reduction” (item 11, the range of scores is minimum 1, maximum 7). Higher scores indicate greater intensity of each smoking effect with, for example, greater satisfaction or psychological reward after vaping.

#### Outcomes

The primary outcome was 12-week CARs (abstinence from combustible cigarettes) during the last 4 weeks (weeks 9–12) of the treatment period of 3 months, defined according to CARs 9–12.^46^ Rates were verified not only by self-reported declarations and study diaries but also by means of exhaled carbon monoxide (≤10 ppm) measurements.^2,47^ Participants who met these criteria were referred to as people who quit.

Secondary outcomes were:

1. Continuous smoking reduction was defined as a reduction by ≥50% in the number of CPD from weeks 9 to 12 compared with BL. CO levels were measured to verify smoking status and confirm also a possible reduction compared with BL. These participants were referred to as people who reduced. No change was defined as participants with a self-reported no change in tobacco smoking. Participants who failed to meet the above criteria at the final week 12 follow-up visit were categorized as people who continued to smoke.
2. CARs at week 24 (CARs 9–24) were defined as sustained self-reported abstinence from tobacco smoking for weeks 9–24 with CO levels of ≤10 ppm.^2,47^
3. Continuous ≥50% reduction in the number of CPD at week 24 was defined as sustained self-reported ≥50% reduction in the number of CPD compared with BL from weeks 9 to 24 follow-up visit. CO levels were measured by the Micro CO to verify smoking status and confirm a reduction, in terms of low number, compared with BL.^48^
4. Feasibility and acceptability as measured by participants’ vital signs, weight, psychopathological changes, and reported AEs from a defined list of side effects. They were also asked to keep track of any AEs they felt may be associated with e-cigarette using the study diary. Subjective effects of using the e-cigarette were assessed by scoring of mCEQ; adoption rate and adherence to e-cigarette use were also assessed. Differences between BL (visit 1) and week 12 (visit 4) for vital signs (blood pressure [BP], heart rate [HR]) and weight were measured. Changes in positive and negative symptoms of schizophrenia were measured by the SANS and SAPS between BL (visit 1) and week 12 (visit 4).

Acceptability of e-cigarettes as substitutes for cigarettes was also assessed; the adoption rate and adherence to product use were measured by (1) daily use of e-cigarettes during the 12 weeks of observation, and (2) ≥50% e-cigarette use during the 12 weeks of observation, which equals at least 42 days of usage. Participants were asked to keep track of the amount they used their e-cigarette using a study diary. The diary was reviewed with the participant by the researcher at each study visit.

For a table of measures and time points, see Table 1.

**Table 1.** Measures and Related Time Points

	Baseline (visit 1)	Week 4 (visit 2)	Week 8 (visit 3)	Week 12 (visit 4)	Week 24 (visit 5)
Primary outcomes					
12-Week continuous abstinence for weeks 9–12			x	x	
Secondary outcomes					
Sustained smoking reduction for weeks 9–12			x	x	
24-Week continuous abstinence for weeks 9–24			x	x	x
Sustained smoking reduction for weeks 9–24			x	x	x
Vital signs (BP and HR)	x	x	x	x	
Weight	x	x	x	x	
SANS and SAPS	x	x	x	x	
Adoption rate and adherence to product use		x	x	x	
AEs	x	x	x	x	
mCEQ	x	x	x	x	
CO	x	x	x	x	x
FTCD	x				

AEs = adverse events; BP = blood pressure; CO = exhaled carbon monoxide; FTCD = Fagerstrom test for cigarette dependence; HR = heart rate; mCEQ = Modified Cigarette Evaluation Questionnaire; SANS = Scale for the Assessment of Negative Symptoms; SAPS = Scale for the Assessment of Positive Symptoms.

### Data Analysis

Primary and secondary outcome measures were computed by including all enrolled participants assuming that, on the basis of the intention-to-treat principle, all individuals lost to follow-up would be classified as people who continued to smoke.^48^ Parametric and nonparametric data were expressed as mean (±SD) and median, interquartile range (IQR) respectively. The proportion of people who quit and people who reduced were reported descriptively. The change in the number of CPD for each visit compared with BL was assessed with the Wilcoxon signed rank test. No adjustments for multiple comparisons were performed given the exploratory nature of this study.

Feasibility and acceptability measures (vital signs and weight, SAPS, SANS, AEs, Serious Adverse Events [SAEs], mCEQ) were reported descriptively. Descriptive statistics were calculated for patient demographics. All *p* values were two-sided with statistical significance evaluated at the .05 alpha level and 95% confidence interval. Analyses were performed in SPSS Version 23 (Statistical Package for Social Sciences Program, IBM Corp., Armonk, NY).

## Results

### Participant Characteristics

**Figure 2.**
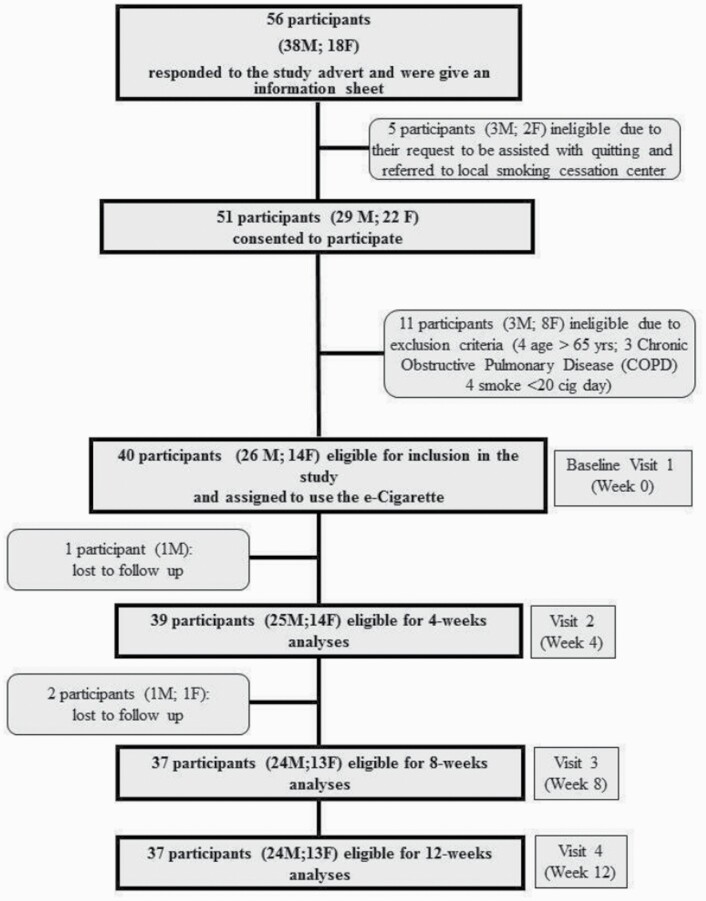
Study flowchart.

Fifty-six adults with schizophrenia spectrum disorders who smoked cigarettes responded to the study advert. A total of 40 participants (M 26; F 14; mean [±SD] aged 48.3 [±12.1] years) adults who smoked (mean [±SD] pack/years of 45.4 [±23.9]) consented to participate and were included in the study (Table 2 and Figure 2). The 40 participants were recruited over a 2-month period. BL characteristics of those who were lost to follow-up were not significantly different from participants who completed the study. Recruitment and 12-week follow-up data are shown in the study flowchart (Figure 2).

**Table 2.** Participants’ Characteristics

	*n* (%)
Sociodemographic characteristics at baseline	
Gender	
Male	26 (65)
Female	14 (35)
Age: mean (SD)	48.3 (12.1)
Age range	
18–24	1 (2.5)
25–44	13 (32.5)
45–65	26 (65)
Education: *n* (%)	
Middle school	22 (55)
High school	17 (42.5)
University	1 (2.5)
Ethnicity	
White Caucasian	40 (100)
Smoking history	
CPD: mean (SD)	28 (9)
Age of onset of smoking: mean (SD)	15.4 (1.2)
Length of time smoking: mean (SD)	33.5 (12.2)
Pack/years: mean (SD)	45.4 (23.9)
Smokers who have made previous cessation attempts: *n* (%)	14 (35%)
Smokers who had previously used an e-cigarette, either regularly or tried: *n* (%)	12 (30%)
FTCD: mean (SD)	8.3 (1.8)
Mental health history and status	
Age onset of schizophrenia spectrum disorders: mean (SD)	21.9 (2.8)
SAPS at baseline: mean (SD)	42.9 (23.7)
SANS at baseline: mean (SD)	43.3 (21.7)

CPD = cigarettes per day; FTCD = Fagerstrom test for cigarette dependence; SANS = Scale for the Assessment of Negative Symptoms; SAPS = Scale for the Assessment of Positive Symptoms.

### Changes in Smoking Behavior

Participants’ tobacco consumption at BL and at 12 weeks is shown in Table 3.

**Table 3.** Participants’ Traditional Cigarette Consumption at Baseline and at 12 and 24 Weeks

Time point	*N* Obs	Variable	Mean	SD	Median
Baseline (visit 1)	40	CPD	27.95	9.14	25.00
		CO	34.03	10.95	30.00
Baseline (visit 1)	37	CPD	28.59	9.79	25.00
Participants who completed the study		CO	33.23	10.97	30.00
Week 12 (visit 4)	37	CPD	6.38	6.89	6.00
		CO	8.19	6.53	10.00
Week 24 (visit 5)	37	CPD	6.91	6.77	7.00
		CO	9.29	8.61	10.00

CO = exhaled carbon monoxide; CPD = cigarettes daily.

### Smoking Cessation

At the 12-week study visits 16/40 (40%) people had quit smoking, with 16/16 (100%) still using their JUUL e-cigarette (Table 3 and Figure 2; Supplementary Figure 1). At the final follow-up visit at week 24, 14/40 (35%) people had quit smoking with 14/14 (100%) still using their JUUL e-cigarette by week 24.

### Smoking Reduction

There was a significant decrease in the number of CPD between BL and 12 and 24 weeks follow-up. A ≥50% reduction in the number of CPD compared with BL for the 4 weeks period prior to the week 12 study visit and was found in 21/40 (52.5%) participants, with a median of 25 CPD at BL (IQR 20, 40) decreasing significantly to 10 CPD (IQR 8.5, 15) (*p* < .001) (Supplementary Table 1).

A ≥50% reduction in the number of CPD at week 24 was found in 23/40 (57.5%) (Supplementary Figure 1) participants with a median of 25 CPD at BL (IQR 20, 40) decreasing significantly to 11 CPD (IQR 8.6, 16) (*p* < .001).

### Acceptability

#### Adoption Rate and Adherence to Product Use

All participants that completed the full schedule of visits (*n* = 37) reported using the e-cigarette each day over the 12 weeks, with a median (IQR) amount of e-cigarettes’ pods of one per day over the study duration.

#### Attrition

At week 12, the retention rate was high, with 37 (92.5%) participants completing all study visits and attending their follow-up visit. One participant chose not to participate after week 2 because he wanted to continue to smoke. The same 37 participants attended the final follow-up visit at week 24. Two participants dropped out of the study after week 4 because they did not find the e-cigarette acceptable and reported that it caused them to cough. Participants’ vital signs, weight, and psychopathological changes from BL to week 12 are shown in Supplementary Table 2.

#### Vital Signs (BP and HR), Weight, and Mental Health

The data indicated that participants’ mean systolic and diastolic BP and HR significantly decreased between BL (visit 1) and 12-week follow-up (visit 4) (*p* = <.0001) (Supplementary Table 2). Participants mean weight between BL (visit 1) and 12-week follow-up (visit 4) also significantly decreased (*p* = .0052) (Supplementary Table 2).

Positive and negative symptoms of schizophrenia were not significantly different after using e-cigarettes from BL (visit 1) to week 12 (visit 4), suggesting absence of psychopathological exacerbation during the period when participants were e-cigarettes. (Supplementary Table 2).

#### Adverse Events

Reported AEs among participants were dry cough (7.8%), headache (5.4%), and throat irritation (2.7%). These events were rare and typical withdrawal symptoms of smoking cessation were not reported. Moreover, there were no reported SAEs during the study.

#### Subjective Effects

The majority of participants (61.9%) were satisfied with using the e-cigarette and obtained psychological reward (85%) that means e-cigarette’ usage calm down, users feel more awake, less irritable, helped with concentration, and reduce hunger for food. The majority of participants (77.5%) reported no aversion to the use of the e-cigarette use and moderate to significant enjoyment (76.1%). 30.1% also reported a moderate craving reduction associated with e-cigarette use (Supplementary Table 3).

## Discussion

In this single-arm study, we assessed whether participants with schizophrenia spectrum disorder could be recruited, examined the feasibility of offering an e-cigarette, assessed changes to smoking behavior, and evaluated the acceptability of an e-cigarette. Participants were recruited within a 2-month period and the majority (92.5%) completed the study. At week 12, 16 of the 40 (40%) participants enrolled in study stopped smoking completely and continued to use the e-cigarette product provided. At week 24, 14 of the 40 (35%) participants enrolled in the study completely stopped smoking and continued to use the e-cigarette product provided. This proportion of people who quit smoking is higher than that found in previous studies with similar patient groups. For example, in the first ever study of e-cigarettes with patients with schizophrenia spectrum disorders,^22^ at week 52, two of 14 (14%) people who smoked at least 20 CPD stopped smoking completely with an e-cigarette. In a study in the United States,^23^ two of 19 (10.5%) participants with serious mental illness switched completely to e-cigarettes at the 4-week assessment. In a more recent study conducted in United Kingdom by Hickling et al.,^24^ in 50 people with severe mental illness who smoked, by the end of the e-cigarette supply phase at week 6, three of 50 participants (7%) reported having stopped smoking tobacco cigarettes.

In week 24 (the end) of this study, a further 57.5% of the participants (23 of 40) were able to sustain ≥50% cigarette reduction by continuing to use the e-cigarette provided. This finding is similar to that in previous studies with similar patient groups. For example, in Caponnetto et al. mentioned above,^22^ at week 52, seven of 14 (50%) participants were able to sustain ≥50% cigarette reduction, verified by a significant reduction in CO levels by 12 months. Pratt et al.^23^ reported a 65% reduction in cigarette use in their participants. In the study by Hickling et al.,^24^ by the end of the e-cigarette supply phase 37% of participants had reduced the number of CPD by ≥50%.

These preliminary findings are promising in view of the fact that all participants in the study were, by eligibility criteria, not motivated to quit smoking. Moreover, although it is not directly comparable with standard smoking cessation and/or reduction studies because of its design, success rates in the present study are not only similar to those obtained with approved pharmaceutical products,^49,50^ but also greater than those observed for first-generation and second-generation e-cigarettes with similar patient groups.^22–24^

This study showed that use of a new-generation e-cigarette, with a high nicotine content, in participants not motivated to quit, substantially decreased cigarette consumption without causing significant side effects. Although not specifically measured in this study, nicotine absorption using new-generation devices has been shown to be consistently superior compared with the first e-cigarette generation devices,^51,52^ which may form part of the explanation for a higher quit rate than in studies using earlier generation devices. Other factors potentially contributing to the higher quit rates compared with previous studies might include the ease of use and durability of the e-cigarette device. This is in marked contrast to one of our earlier studies^22^ in which participants used a five-piece e-cigarette product that was complicated to use (Supplementary Figure 2). For example, in that study, batteries, chargers, and atomizing devices frequently broke, and cartridges often leaked liquid into the mouth and needed frequent changing. Therefore, avoiding unnecessary stress to the participants may also have influenced acceptability and adherence. Participants’ responses to the mCEQ suggest they found the JUUL satisfying and enjoyable, and this likely enhanced adherence. Given its technological advantages, as well as its pharmacokinetic profile similar to that of combustible tobacco cigarettes,^28,29,53,54^ the e-cigarette used in this study may have provided a more “cigarette-like” experience which may have potentially stimulated switching, although this was not formally assessed.

The study procedures appeared acceptable to participants. This is evidenced by the high retention rate. Given that few problems were experienced in conducting the research, the study procedures were also feasible. There is also evidence of acceptability in terms of the short-term markers of physical and mental health assessed during the study. Participants showed significant improvements in BP and HR, did not gain weight, and exhibited no exacerbation in psychopathology. Other studies suggest that symptoms of schizophrenia are not increased in patients who reduce their smoking using nicotine patches^55,56,57^ or early generation e-cigarettes.^22^ However, a minority of study participants reported the administration of SAPS and SANS at each visit was too long, and so this needs to be taken into consideration for future studies.

There were several limitations in this study. First, this was an uncontrolled study and the lack of a control group and blinding limits the internal validity and external validity of the findings. Confounding is a major threat to the validity of uncontrolled data. Thus, this underscores the need for a future RCT. Nonrandomized or single-arm pilot studies are an acceptable approach to testing the feasibility of interventions.^32^ These types of studies constitute research in which all or part of an intervention is evaluated and other study procedures are piloted for a future definitive trial, but without randomization of participants. These studies are used to determine whether an intervention is appropriate for further testing. Performing this kind of study may be indicated when there are few previously published studies or existing data using a specific intervention technique. Commonly a number of issues are examined in these studies: initial efficacy, acceptability, demand, implementation, practicality, and adaption.^32,55,56^ Given the very limited existing literature on the use of e-cigarettes for smoking reduction or cessation in adults with schizophrenia and no literature on use of a high strength nicotine e-cigarettes, it was decided that a single-arm pilot study should be the first step to gather data to inform the design of a future trial.

Characteristics of the sample and the e-cigarette used limit the generalizability of the findings. We enrolled participants who smoked at least 20 CPD and who were unwilling to quit, and therefore our findings cannot be assumed to apply to all adults with schizophrenia who smoke cigarettes. Specifically, direct comparison with other smoking cessation studies involving individuals who smoke—and who are motivated to quit—cannot be made. All participants were from urban Sicilian outpatients settings and may not be valid for other population samples. Lastly, because only a single nicotine strength (ie, 5%) and a single flavor (ie, Virginia tobacco) were investigated in this study, it is possible that the study did not provide options that could have increased acceptability and cessation success rates. Other research has suggested that unrestricted access to a wider selection of e-liquid nicotine strengths and flavors can play an important role in the attractiveness and success rates of these products.^58^

In conclusion, preliminary positive results from this single-arm pilot study suggest that this intervention could be tested in an RCT. It will be important to discard or modify those parts of the assessment procedures that participants reported were burdensome, such as the duration of time required for administration of SAPS and SANS at each visit. Many participants also wanted to use the e-cigarette for a longer period of time. In addition to this, participants requested more counseling about how to give up smoking and vaping; hence, a specific brief smoking cessation counseling intervention could be added to help individuals who smoke to switch first to an e-cigarette and then progressively become totally smoke and vape free. A future RCT could also include study sites in other countries to improve generalizability.

In summary, this study aimed to conduct preliminary research to begin to determine the efficacy of a high strength nicotine e-cigarette in a group of people with schizophrenia spectrum disorders who smoked cigarettes. The results provide useful information and direction to add to the existing body of knowledge on smoking cessation for this group.

## Supplementary Material

A Contributorship Form detailing each author’s specific involvement with this content, as well as any supplementary data, are available online at https://academic.oup.com/ntr.

ntab005_suppl_Supplementary_Figure_1

ntab005_suppl_Supplementary_Figure_2

ntab005_suppl_Supplementary_Table_1

ntab005_suppl_Supplementary_Table_2

ntab005_suppl_Supplementary_Table_3

ntab005_suppl_Supplementary_Taxonomy_Form
