# Supplementary material for: A Single-Arm, Open-Label, Pilot, and Feasibility Study of a High Nicotine Strength E-Cigarette Intervention for Smoking Cessation or Reduction for People With Schizophrenia Spectrum Disorders Who Smoke Cigarettes
Source: Nicotine Tob Res. 2021 Mar 16;23(7):1113–22. doi: 10.1093/ntr/ntab005 (PMC8186418; doi:10.1093/ntr/ntab005)
Supplement: ntab005_suppl_Supplementary_Figure_1 [file ntab005_suppl_supplementary_figure_1.docx]

**Supplementary figure 1. Changes in smoking behaviour at week 12**
