# Supplementary figures and images for: A Single-Arm, Open-Label, Pilot, and Feasibility Study of a High Nicotine Strength E-Cigarette Intervention for Smoking Cessation or Reduction for People With Schizophrenia Spectrum Disorders Who Smoke Cigarettes
Source: Nicotine Tob Res. 2021 Mar 16;23(7):1113–22. doi: 10.1093/ntr/ntab005 (PMC8186418; doi:10.1093/ntr/ntab005)

**Supplementary figure 2. Five-piece e-cigarette vs Juul**


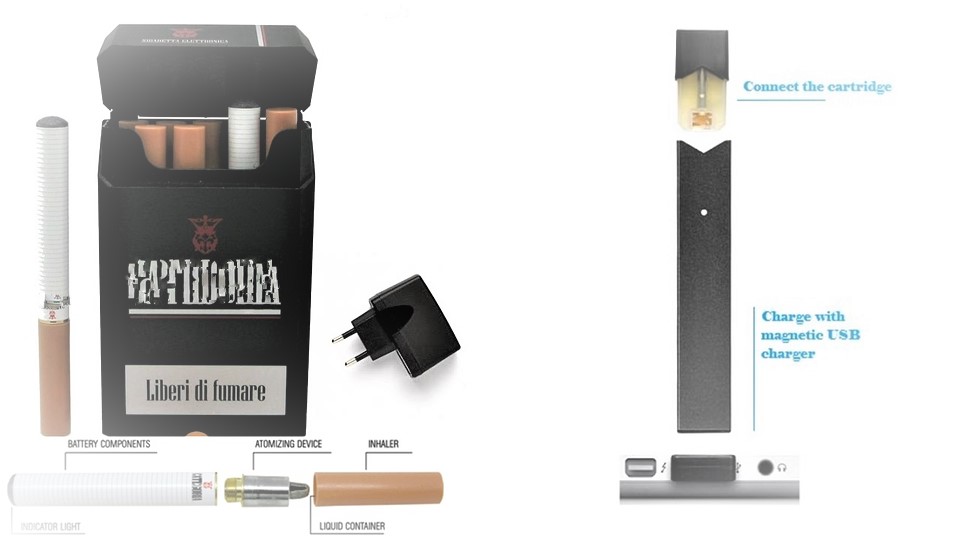

Supplement: ntab005_suppl_Supplementary_Figure_2 [file ntab005_suppl_supplementary_figure_2.docx]
