# Supplementary material for: A Single-Arm, Open-Label, Pilot, and Feasibility Study of a High Nicotine Strength E-Cigarette Intervention for Smoking Cessation or Reduction for People With Schizophrenia Spectrum Disorders Who Smoke Cigarettes
Source: Nicotine Tob Res. 2021 Mar 16;23(7):1113–22. doi: 10.1093/ntr/ntab005 (PMC8186418; doi:10.1093/ntr/ntab005)
Supplement: ntab005_suppl_Supplementary_Table_1 [file ntab005_suppl_supplementary_table_1.docx]

**Table 4: Smoking behaviour outcomes**

| **Parameter** | **Baseline** | **12 weeks** | ***p-value‡*** |
| --- | --- | --- | --- |
| Quitters. 100% reduction in cigarette smoking (n=16) Age  Pack Years  CPD | 47.3(±9.7)†  32.1(±14.5)†  20 (20, 30)* | 0 (0, 0)* | <0,001 |
| Reducers. 50% reduction in cigarette smoking (n=21)  Age  Pack Years  CPD | 49.3 (±14.1)†  54.4 (±23.6)†  25 (20,40)* | 10 (8.5, 15)* | <0.001 |

CPD; cigarettes daily; IQR: interquartile range; SD: standard deviation

‡ p-value within group Wilcoxon signed-rank test

† Parametric data expressed as mean (±SD)

*Non-parametric data expressed as median (IQR)
