# Supplementary material for: A Single-Arm, Open-Label, Pilot, and Feasibility Study of a High Nicotine Strength E-Cigarette Intervention for Smoking Cessation or Reduction for People With Schizophrenia Spectrum Disorders Who Smoke Cigarettes
Source: Nicotine Tob Res. 2021 Mar 16;23(7):1113–22. doi: 10.1093/ntr/ntab005 (PMC8186418; doi:10.1093/ntr/ntab005)
Supplement: ntab005_suppl_Supplementary_Table_2 [file ntab005_suppl_supplementary_table_2.docx]

**Table 5. Participants’ vital signs, weight and psychopathological changes from baseline to week 12**

| **Variables overall group** | **Mean BL** | **SD**  **BL** | **Median**  **BL** | **Mean WK12** | **SD**  **WK12** | **Median**  **WK12** | **p-value** |
| --- | --- | --- | --- | --- | --- | --- | --- |
| weight heart rate bp systolic bp diastolic sans saps | 75.98 80.13 133.48 76.73 43.33 42.90 | 14.56 9.32 9.91 7.20 21.76 23.74 | 73.75 79.50 135.00 80.00 41.00 36.50 | 74.67 72.29 121.41 70.30 44.89 43.16 | 14.22 6.19 8.59 4.75 21.33 24.91 | 71.00 73.00 123.00 70.00 44.00 36.00 | 0.0052  <0.0001  <0.0001  <0.0001  0.932  0.809 |
| **Variables Quitters** |  |  |  |  |  |  |  |
| weight heart rate bp systolic bp diastolic sans saps | 76,27 77,75 131.06 76.19 45,19 42.90 | 18.18 11.51 11.48 8.23 26.54 25.73 | 70.00 76.50 133.50 79.00 30.00 41.25 | 74.40 69.12 120.81 68.75 47.81 45.00 | 16.27 5.46 9.47 5.53 26.45 25.64 | 70.00 69.00 124.00 70.00 39.00 38.50 | 0.012  <0.0001  0.003  0.001  0.317  0.655 |
| **Variables Reducers** |  |  |  |  |  |  |  |
| weight  heart rate  bp systolic  bp diastolic  sans  saps | 75.32 82.14 134.24 76.67 40.24 38.90 | 12.96 7.23 8.81 6.95 19.81 21.85. | 73.75 80.00 135.00 80.00 37.00 40.00 | 75.05 75.71 121.80 71.47 41.23 38.61 | 12.63 4.60 8.10 3.78 18.83 21.83 | 74.00 75.00 122.00 70.00 43.00 36.00 | 0.154  <0.0001  <0.0001  0.003  0.686  0.500 |
