# Supplementary material for: A Single-Arm, Open-Label, Pilot, and Feasibility Study of a High Nicotine Strength E-Cigarette Intervention for Smoking Cessation or Reduction for People With Schizophrenia Spectrum Disorders Who Smoke Cigarettes
Source: Nicotine Tob Res. 2021 Mar 16;23(7):1113–22. doi: 10.1093/ntr/ntab005 (PMC8186418; doi:10.1093/ntr/ntab005)
Supplement: ntab005_suppl_Supplementary_Table_3 [file ntab005_suppl_supplementary_table_3.docx]

**Supplementary Table 3. Subjective effects of e-cigarettes at** **Week 12: Participant responses to mCEQ questions n (%)**

| **"SMOKING SATISFACTION"** | 1  not at all | 2  very little | 3  a little | 4  moderately | 5  a lot | 6  quite a lot | 7  extremely |
| --- | --- | --- | --- | --- | --- | --- | --- |
| Q1 “Was using your e-cigarette satisfying? | 2  (1.8) | 6  (5.3) | 20  (17.7) | 16  (14.2) | 46  (40.7) | 19  (16.8) | 4  (3.5) |
| Q2 “Does the e-cigarette taste good? | 4  (3.5) | 4  (3.5) | 7  (6.2) | 28  (24.8) | 58  (51.3) | 11  (9.7) | 1  (0.9) |
| Q12 “Do you enjoy using your e-cigarette?” | 1  (0.9) | 2  (1.8) | 12  (10.6) | 28  (24.8) | 44  (38.9) | 15  (13.3) | 11  (9.7) |
| **"PSYCHOLOGICAL REWARD"** | 1  not at all | 2  very little | 3  a little | 4  moderately | 5  a lot | 6  quite a lot | 7  extremely |
| Q4 “Does using your e-cigarette calm you down?” | 14  (12.4) | 10  (8.8) | 38  (33.6) | 22  (19.5) | 20 (17.7) | 8  (7.1) | 1  (0.9) |
| Q5 “Does using your e-cigarette make you feel more awake?” | 25  (22.1) | 20  (17.7) | 36 (31.9) | 18  (15.9) | 13 (11.5) | 1  (0.9) | 0  (0.0) |
| Q6 “Did using your e-cigarette make you feel less irritable?” | 16  (14.2) | 18  (15.9) | 33 (29.2) | 26  (23.0) | 12 (10.6) | 8  (7.1) | 0  (0.0) |
| Q7 “Did using your e-cigarette help you concentrate?” | 21  (18.6) | 13  (11.5) | 41 (36.3) | 16  (14.2%) | 17 (15.0) | 5  (4.4%) | 0  (0.0%) |
| Q8 “Does using your e-cigarette reduce your hunger for food?” | 33  (29.2) | 32  (28.3) | 22 (19.5) | 18  (15.9) | 6  (5.3) | 2  (1.8) | 0  (0.0) |
| **“AVERSION”** | 1  not at all | 2  very little | 3  a little | 4  moderately | 5  a lot | 6  quite a lot | 7  extremely |
| Q9 “Does using your e-cigarette make you dizzy?” | 92  (81.4) | 13  (11.5) | 5  (4.4) | 0  (0.0) | 3  (2.7) | 0  (0.0) | 0  (0.0) |
| Q10 “Does using your e-cigarette make you nauseous?” | 83  (73.5) | 15  (13.3) | 13 (11.5) | 1  (0.9) | 0  (0.0) | 0  (0.0) | 1  (0.9) |
| **“ENJOYMENT OF RESPIRATORY TRACT SENSATIONS"** | 1  not at all | 2  very little | 3  a little | 4  moderately | 5  a lot | 6  quite a lot | 7  extremely |
| Q3 “Do you enjoy the sensations in your throat and chest?” | 11  (9.7) | 2  (1.8) | 14 (12.4) | 34  (30.1) | 32 (28.3) | 14 (12.4) | 6  (5.3) |
| **“CRAVING REDUCTION”** | 1  not at all | 2  very little | 3  a little | 4  moderately | 5  a lot | 6  quite a lot | 7  extremely |
| Q11 “Does using your e-cigarette immediately reduce your craving for nicotine?” | 6  (5.3) | 5  (4.4) | 23 (20.4) | 34  (30.1) | 23  (20.4) | 18 (15.9) | 4  (3.5) |
